# The Genetics of Chiari 1 Malformation

**DOI:** 10.3390/jcm13206157

**Published:** 2024-10-16

**Authors:** Rachel E. Yan, John K. Chae, Nadia Dahmane, Palma Ciaramitaro, Jeffrey P. Greenfield

**Affiliations:** 1 Department of Neurological Surgery, Weill Cornell Medicine, New York, NY 10065, USA; ray4001@med.cornell.edu (R.E.Y.); nhc9003@nyp.org (J.K.C.); nad2639@med.cornell.edu (N.D.); 2 Neuroscience Department, Azienda Ospedaliera-Universitaria Città della Salute e della Scienza di Torino, 10126 Torino, Italy; palma.ciaramitaro@gmail.com

**Keywords:** Chiari malformation, genetics, familial aggregation, sequencing, posterior fossa, cerebellum

## Abstract

Chiari malformation type 1 (CM1) is a structural defect that involves the herniation of the cerebellar tonsils through the foramen magnum, causing mild to severe neurological symptoms. Little is known about the molecular and developmental mechanisms leading to its pathogenesis, prompting current efforts to elucidate genetic drivers. Inherited genetic disorders are reported in 2–3% of CM1 patients; however, CM1, including familial forms, is predominantly non-syndromic. Recent work has focused on identifying CM1-asscoiated variants through the study of both familial cases and de novo mutations using exome sequencing. This article aims to review the current understanding of the genetics of CM1. We discuss three broad classes of CM1 based on anatomy and link them with genetic lesions, including posterior fossa-linked, macrocephaly-linked, and connective tissue disorder-linked CM1. Although the genetics of CM1 are only beginning to be understood, we anticipate that additional studies with diverse patient populations, tissue types, and profiling technologies will reveal new insights in the coming years.

## 1. Introduction

**Figure 1:**
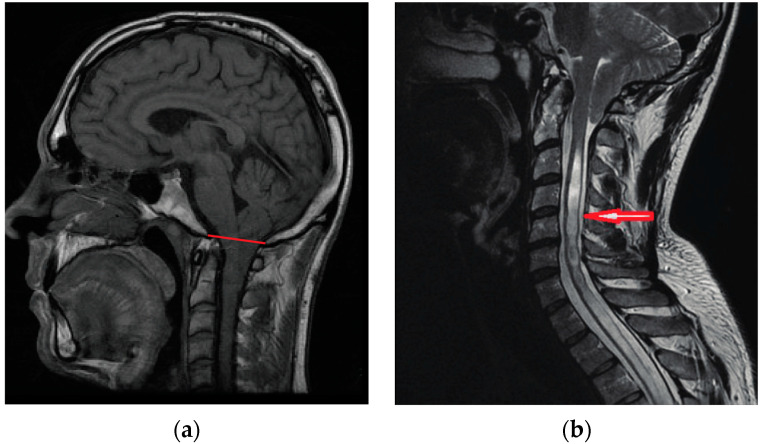
Chiari 1 Malformation (CM1). Sagittal MRI (**a**) Male, 24 years; CM1 is defined as a displacement of the cerebellar tonsils greater than five millimeters below the basion–opisthion line (red line); (**b**) Female, 38 years; CM1 with long segment syringomyelia (red arrow).

Chiari malformation type 1 (CM1) is characterized by a caudal displacement of the cerebellar tonsils below the foramen magnum at the base of the skull [1,2], and is classified among cerebellar dysplasias (Q07; ORPHA268882). It is diagnosed primarily by magnetic resonance imaging (MRI) and is defined as the herniation of the cerebellar tonsils greater than five millimeters below the basion–opisthion line (i.e., McRae line) (Figure 1a). Many cases often also have skull morphometric variations, such as short clivus and craniovertebral junction [3]. Symptomatology varies between patients and severity is not necessarily correlated with the extent of tonsillar displacement [2,4]. According to epidemiological data, the prevalence of CM1 meeting imaging criteria is 7/100,000 of which 4.5/100,000 (58%) are asymptomatic [5,6,7]. Therefore, clinical history and features (neurological symptoms and signs) complete the clinical diagnosis of CM1. It is likely that brain compression and cerebrospinal fluid disturbances underlie symptoms. When symptoms persist, treatment aimed at symptom amelioration is primarily surgical, involving the decompression of the posterior fossa.

Over the years, Chiari malformations have been associated with a number of potential etiologic causes leading to tonsillar herniation, involving developmental mechanisms and anatomic consequences. Morphometric studies (reviewed by Shuman et al. in [3]) have found alterations in the posterior fossa, cervical spine, and cerebellum/hindbrain, such as reduced clivus length [3]. Many cases have also been linked with co-occurring syndromes, including those affecting the skull (e.g., craniosynostosis [7,8,9], connective tissue disorders (e.g., Ehlers Danlos [10,11,12]), and developmental disorders (e.g., Cowden syndrome [13])). Recent studies have also expanded the repertoire of associated genetic alterations using SNP panels (e.g., [14,15,16,17,18,19,20,21]) and sequencing (e.g., [22,23,24,25,26,27,28]) of individuals, family members, and larger cohorts. Here, we review the current literature on the genetics of CM1, and briefly discuss future directions.

## 2. Evidence for a Heritable Genetic Basis

Throughout the years, CM1 has been associated with many inherited genetic disorders. Though typically sporadic, familial cases have been reported [29,30]. Even without co-occurring disorders, CM1 tends to aggregate within families. In a large series of 364 patients, it was found that 12% of CM1 cases aggregated within families with a Mendelian-like inheritance pattern [2]. Familial aggregation has also been reported in over 38 reports in the literature. Among twin studies, including 17 pairs of twins and one set of triplets, 13 were concordant with CM1 (76%) [31,32,33,34,35,36,37,38,39,40,41,42,43,44]. The high concordance provides clear evidence for a heritable cause of CM1, which is likely influenced by environmental factors that may exacerbate an existing predisposition to tonsillar ectopia. Rare prenatal cases have been reported [45]. There is also a significantly increased incidence in women (75–80%), as seen in two prospective studies of symptomatic CM1 patients [2,7], which may be related to a smaller posterior fossa volume in women [46].

## 3. An Anatomic Defect

### 3.1. Defects in Posterior Fossa Morphology

The posterior fossa contains the cerebellum, pons, and medulla. It is bordered posteriorly by the occipital bone, laterally by the two temporal bones, and superiorly by the tentorium. The brainstem transitions into the spinal cord as it exits the posterior fossa through the foramen magnum, a large opening at the base of the skull. While the cerebellum is normally contained within the posterior fossa, the cerebellar tonsils herniate through the foramen magnum in CM1 (Figure 1).

The skull base and vertebral column develop in concert with the inductive effects of the brain and notochord [47]. The posterior skull base originates from the mesoderm, in contrast to the cerebellum, pons, and medulla, which are derived from the ectoderm [48,49,50]. The posterior skull base begins its development when the rostral tip of the notochord reaches just caudal to the hypophysis. The notochord’s sulfated glycosaminoglycans induce the chondrification of occipital sclerotome-derived mesenchyme, which ossifies to the basioccipital elements of the occipital bone by week 7, followed by the chondrification of the exoccipital elements [51]. 

Because the cerebellum rests in the posterior fossa and is limited by its bony confines, it has been hypothesized that a small posterior fossa may cause the tonsillar herniation present in CM1 [52]. Supporting this, the median cross-sectional area of the posterior fossa (sagittal) is approximately 23% smaller in patients with CM1, with significant decreases in both the anterior–posterior (AP) dimension and the width [53,54,55]. Despite the convincing etiology of a small posterior fossa, it may not be the cause of all Chiari malformations [56]. Many patients have CM1 without reduced posterior fossa volume [4,57], including familial cases [58]. An underdeveloped posterior fossa cannot explain Chiari malformations occurring before midgestation [59]. A large foramen magnum may also facilitate CM1 development by facilitating cerebellar herniation, as well as decreased intraspinal pressure or other weaknesses in tissue. A large cerebellum, or other geometric constraints on space, may also facilitate herniation. Furthermore, alterations in posterior fossa morphology resulting in compression may be more significant than the herniation itself. Lesions causing tonsillar herniation (“false tonsils descent”, “acquired tonsillar ectopia” or “acquired Chiari malformation”), such as tumors or hydrocephalus may also occur, but should be considered separate from their familial counterpart [60], and will not be covered in this review. In the next sections, we will consider several anatomic and developmental etiologies of CM1, and link them to potential molecular mechanisms.

### 3.2. Defects in Posterior Fossa Development

Developmental defects in posterior fossa morphology may be linked to CM1. In the following paragraphs, we summarize several genes and pathways related to posterior fossa morphology and potentially CM1.

A. RA/FGF signaling

In 1981, Marin-Padilla and Marin-Padilla demonstrated one potential mechanism for CM1 development using a hamster model [61]. By administering a high dose of vitamin A to pregnant hamsters on the eighth day of gestation, they were able to induce CM1 in their offspring. Specifically, their offspring had a CM1-like phenotype with displaced cerebellar tonsils, a small posterior fossa, a short basichondrocranium, and an underdeveloped occipital bone [61]. Accordingly, CM1 has also been reported in patients with mutations in the *retinoic acid receptor beta* (*RARB*) [62]. Retinoic acid is a metabolite of dietary vitamin A with many roles in embryonic development and cellular differentiation [63]. Furthermore, retinoic acid regulates fibroblast growth factor (FGF) during development [64]. As with retinoic acid signaling, *FGFR3* gain-of-function mutations result in defects in posterior fossa morphology in mouse models, including alterations in the foramen magnum shape and size [65]. *FGFR* mutations are also linked with other disorders, such as craniosynostosis and achondroplasia.

Craniosynostosis is a premature closure of one or more sutures between the skull plates and is often linked with CM1 (present in 1–27% of the cases of CM1; CM1 in 50–100% of craniosynostosis) [7]. The premature closure of the sutures may affect the posterior fossa, which may lead to overcrowding and tonsillar herniation [66]. Craniosynostosis can occur as a part of syndromes, including Crouzon syndrome [66,67,68,69,70], Muenke syndrome [71,72], Pfeiffer syndrome type 2 [67,73] and 3 [24], and others [74,75,76], which have also been linked to CM1. Specifically, recurrent mutations associated with FGF signaling in craniosynostosis may also be associated with CM1. Reports in the literature include seven cases of p.Ala391Glu *FGFR3*, three cases of p.Pro250Arg *FGFR3*, two cases of p.Cys342Trp *FGFR2*, and two cases of p.Tyr375Cys [66,67,68,69,75,76]. Specific investigations into *FGFR2*-induced craniosynostosis have suggested that even among cases with *FGFR*-linked mutations, the mechanism for Chiari pathogenesis may differ [77].

In addition to craniosynostosis, mutations in *FGFR3* are also associated with achondroplasia, which has also been linked to CM1 [12,78]. Achondroplasia is the most frequent cause of dwarfism and is due to defects in endochondral ossification [65]. In achondroplasia, *FGFR3* mutations cause disruptions in the ossification of the skull base, thus affecting the shape and size of the foramen magnum and posterior fossa, explaining the Chiari phenotype.

Recurrent mutations in *FGFR2* and *FGFR3* have also been found in patients with CM1 without either of these associated disorders [67,74]. Though patients with mutations in *FGFR*s tend to present with either craniosynostosis or achondroplasia, it is possible that some of these alterations are specifically associated with CM1 [68].

B. Additional pathways

In addition to RA and FGF signaling, other genes involved in similar processes may also play a role. For example, there have been at least seven individual cases of associated mutations with CM1 and craniosynostosis in the *ETS2 Repressor Factor* (*ERF)* gene [8,9,79,80]. ETS2 may also act downstream of FGF signaling in mesodermal induction [81]. Small posterior fossa may also be associated with other cranio-cervical bone anomalies such as occipital dysplasia, which commonly leads to an underdeveloped occipital bone [53], and pseudoparathyroidism type 1A, which may lead to small posterior fossa size [82]. Recently, Brockmeyer et al. [83] built on data from Abbott et al. [30] utilizing the Utah Population Database to identify a candidate variant in *HOXC4* that may affect the development of the junction between the mesoderm and neural crest, leading to decreased clivoaxial angle and CM1 [83]. Matsuoka et al. proposed that the widening of the foramen magnum, due to post-otic neural crest-derived mesenchymal stem cells being re-specified from bone to connective tissue, may cause CM1 [84]. A similar phenotype has also been found in mice heterozygous for *Suz12*, a gene encoding for a member of the polycomb repressive complex 2 (PRC2) that is involved in epigenetic gene regulation [85]. This widened foramen magnum is similar to the anatomic defect that may be seen in achondroplasia [65].

From an evolutionary perspective, it has been proposed that CM1 may be related to the evolution of human bipedalism [86] because of the change in the angle between the skull and spine requiring the shortening of the basioccipital region [87]. This shrinkage leads to a significant reduction in the volume of the posterior fossa (27%), which shrinks an additional 21% in Chiari patients [54,87]. While it is unclear which mechanisms lead to this change, it is likely that the genes or regulatory effects leading to this change may be similarly altered in CM1.

### 3.3. Defects in Brain Growth

In addition to skull morphology (the ‘container’), the brain itself (e.g., macrocephaly) may contribute to the development of CM1 due to overcrowding within the cranial space [52]. Overcrowding may be seen in several syndromic forms, including Costello syndrome [88], macrocephaly-capillary malformation [89], and megalencephaly-polymicrogyria-polydactyly-hydrocephalus [90], all of which involve increased postnatal brain growth. In these syndromes, which are related to Ras-MAPK dysregulation, it is proposed that abnormal neurogenesis or gliogenesis may cause megalocephaly, ventriculomegaly, hydrocephalus, and the progressive herniation of the cerebellar tonsils [44]. Similar mechanisms may be involved with CM1 and other overgrowth syndromes, such as neurofibromatosis type I, neurofibromatosis type II, Sturge–Webe, McCune–Albright, Beckwith–Wiedemann, cutis marmorata telangiectatica congenita, Klippel–Trenaunay, PIK3R2-related overgrowth, CLOVES (Congenital Lipomatous Overgrowth, Vascular Malformations, Epidermal Nevis, Spinal/Skeletal Anomalies/Scoliosis) syndrome, Tatton-Brown–Rahman, Noonan syndrome, Osler–Weber–Rendu, tuberous sclerosis, and Luscan-Lumish syndrome [44,91,92]. This line of evidence also suggests that volumetric mismatch between the cerebellum and the posterior fossa may underly non-syndromic CM1 cases.

Recent sequencing studies have confirmed that mutations in brain growth-related genes may be linked with CM1. Sadler et al. performed whole exome sequencing on 67 parent-offspring trios and identified loss of function mutations in *CHD8*, *CRIM1*, and *ARL8A* [93]. *CHD8*, encoding for a chromodomain containing chromatin remodeler protein, is frequently mutated in autism [94]. Mice with CHD8 haploinsufficiency have increased brain weight [95], defects in neurogenesis [96], and develop brain overgrowth (megaencephaly) [96,97] (reviewed in [98]). Zebrafish with heterozygous loss of CHD8 function also have increased overall brain growth (including the forebrain, midbrain, and hindbrain) [92]. Sadler et al. also extended their analysis to probands without family members and found support for a role of CHD/CHD3 mutations in CM1, linked with increased head circumference [93]. This reinforces the hypothesis that brain overgrowth (likely including several regions) may play a role in the etiology of both syndromic and non-syndromic CM1. Notably, mutations in other chromatin remodeling genes, such as histone methyltransferases, histone demethylases, and histone proteins, were also found in a recent study of 51 patients with CM1 and their relatives [28]. 

### 3.4. Defects in Connective Tissue

In addition to the skull, connective tissue also plays a crucial role in supporting the cerebellum. Boyles et al. also performed a linkage analysis on 23 families with 71 affected individuals [21]. Of the 10,000 SNPs analyzed, they found possible associations with 15q21.1–22.3 and 9q21.33–33.1 [21]. 15q21.1–22.3 contains the *FBN1* gene, which is linked with Marfan syndrome and Shprintzen–Goldberg syndrome, which are known to co-occur with CM1 [21]. Other connective tissue disorders such as Ehlers–Danlos syndrome type 3 (hypermobility type) [92], Loeys–Dietz syndrome (LDS), Stickler syndrome, and self-identified hypermobility [92,99] have also been linked with CM1. This association may be linked to increased tissue mobility and decreased stability, which could facilitate cerebellar herniation. Mutations in *COL4A1* have also been reported in association with CM1 [100]. Thus, each broad anatomic cause of CM1 may be associated with multiple potential genetic mechanisms.

## 4. Additional Results from Large Cohort Studies

A small posterior fossa is highly heritable [21,101], which supports that genetic mechanisms may explain some of the developmental and anatomic hypotheses discussed above. Musolf et al. evaluated this possibility using whole exome sequencing on seven extended families from Russia with a family history of CM1 [27]. Linkage analysis revealed two loci associated with small posterior fossa size at 1q43–44 and 12q23 [27]; however, each of these loci was driven by a single family, thus the generalizability is limited.

In a larger study, Markunas et al. used linkage analysis to study 367 individuals from 66 families with two or more individuals with non-syndromic CM1 [20]. Of the patients without connective tissue disorder-associated symptoms, there was evidence of potential linkage with areas including the *GDF3* and *GDF6* genes, which encode for growth differentiation factors implicated in Klippel–Feil syndrome [20], found in 3–5% of the CM1 patients [2,102]. These findings highlight the relationship between co-occurring genetic disorders and CM1 but note that familial associations with CM1 may be confounded by them.

While a significant proportion of CM1 are familial cases or related to known genetic syndromes, the vast majority are not. In these cases, it is important to consider genetic linkage outside of Mendelian disorders. Using linkage analysis, a case–control association study of SNPs across 58 candidate genes in 404 CM1 patients found linkages to four SNPs in *CDX1*, *FLT1*, and *ALDH1A2* in a subgroup with shallow posterior fossa (*n* = 186) [18]. These findings complement the alternative approach of linkage analysis to posterior fossa hypoplasia in general, which has been linked to loci including 15q21.1–22.3, 8q, 22q, 1q, and 12p [21], though it was limited to only 58 candidate genes.

Lock et al. also performed a linkage analysis, though it focused specifically on expression quantitative trait loci (eQTL), which are polymorphisms affecting gene expression levels [19]. In the blood and dura of 43 patients with CM1, they identified 239 candidates, 79% of which were identified in both tissues. Notable candidates included *IPO8*, *XYLT1*, and *PRKAR1A*, which are associated with osteoblast differentiation, chondrocyte maturation, and bone growth, respectively [19]. Genes associated with ribosome function such as *RPS20/23/26*, *RPL14/36AL*, *NSA2*, and *RPF2* were also commonly altered [19]. The same group, led by Markunas et al. had previously stratified this group into two classes based on k-means clustering, a type of unsupervised learning to discover subgroups within a dataset [103]. Class 1 had upregulated genes related to dorsal–ventral axis formation and cancer-related pathways in the dura, as well as the upregulated ribosome, spliceosome, proteome, RNA degradation, and oxidative phosphorylation genes in the blood in comparison to opposing changes in Class 2 [103]. Class 1 was also characterized by younger paternal age relative to that of Class 2 with differences in radiographic features, which were generally smaller in Class 2 [103]. This highlights heterogeneity within CM1 etiology.

Following the posterior fossa hypothesis, the same group also performed an ordered subset analysis stratified by groups with various heritable posterior fossa traits associated with CM1, allowing them to identify *EP300*, *CREBBP*, and *ATF4* as additional candidates [101]. These genes encode proteins that regulate SOX9, a transcription factor involved in chondrocyte differentiation, and are associated with a disorder that has also been found to co-occur with CM1, Rubenstein–Taybi syndrome. Reported mutations in patients with Rubinstein–Taybi and CM1 have included *CREBBP*, c.3546ins, c.3546insCC, p.R2004X, c.4482dupC, c.4944dupC, and a 520.7 kb microdeletion on 16p13.3 (encompassing *CREBBP*) [37,104,105,106,107]. Another region was also identified including *LHX4*, which was found to be altered in a case report of a patient with combined pituitary hormone deficiency and CM1 (p.P366T *LHX4*) [108]. The recurring association with various co-occurring syndromes continues to suggest that some cases of Chiari may be due to variable expressivity of these syndromes or mosaic cases.

**Figure 2:**
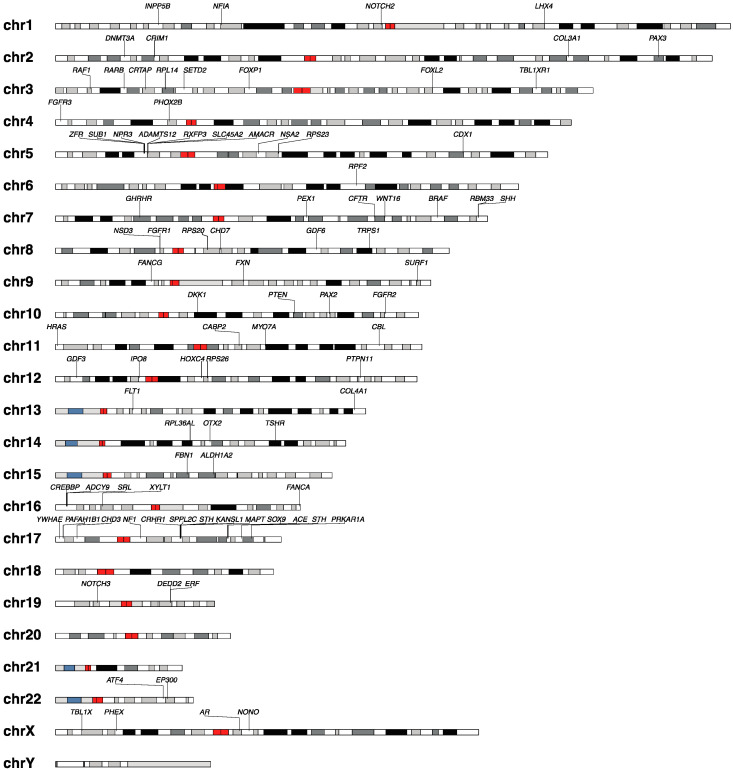
Overview of key genes linked with CM1 and CM1-associated syndromes. Genes were summarized from studies on individual patients, families, and larger cohort analyses [8,9,12,13,14,15,16,17,18,19,20,22,23,24,25,26,28,37,45,62,66,67,68,69,71,72,73,74,75,76,78,79,80,83,88,93,100,104,105,106,107,108,110,111,112,113,114,115,116,117,118,119,120,121,122,123,124,125,126,127,128,129,130,131,132,133,134,135,136,137,138,139,140,141,142,143,144,145,146,147,148,149,150,151,152,153,154,155,156,157,158,159,160,161,162,163]. Genes linked to syndromes associated with CM1 were included. Black/grey: heterochromatin; red: centromere; blue: variable regions [164].

Finally, in a recent study of 51 isolated and syndromic pediatric cases, Provenzano et al. propose that CM1 is a Mendelian trait (autosomal dominant in most cases) [28]. They find that both the syndromic and sporadic cases of CM1 often contain variants in chromatin-remodeling genes, which they further separate into five categories (W1–W5), including *SETD2*, *NSD3*, *SMID3* (W1 category), *KDM5* (W2 category), *EP300*, *CREBBP* (W3 category), *SETBP2* (W4 category), and *HIST1* (W5 category) [28]. Chromatin remodeling plays a central role in modulating gene expression, and has been identified in a number of disorders, including cancer and neurodevelopmental disease [109]. Genes involved in the sutures of the cranial bones, microcephaly, closure of the neural tube (e.g., *VANGLI1*), and in RASopathy (e.g., *BRAF* and *CBL*) were also identified [28]. While the authors did not find any clinical signs that were pathognomonic of a particular gene/variant in sporadic CM1, they were underpowered to detect this. Surgery resulted in radiologic improvements of the CM1, regardless of the causative variant [28]. Of note, mutations have also been found in bone mineral density-related genes using targeted sequencing, such as *WNT16*, *CRTAP*, *MYO7A*, and *NOTCH2* [110]. Altogether, these findings provide interesting hypotheses that will need to be confirmed in larger studies (for an overview of genes, see Figure 2).

## 5. Discussion

In recent years, significant efforts have been made to identify the genetic drivers of CM1 through linkage analysis and the whole exome sequencing of trios. Relationships with other disorders have also been reported throughout history, providing additional mechanistic insight into potential etiologies and related genetic mechanisms. These data have revealed an array of potential genetic drivers, often related to different anatomic etiologies underlying the CM1 phenotype. However, cohort sizes are still limited and thus studies are underpowered.

As tools such as whole exome and whole genome sequencing become increasingly affordable and accessible, the comprehensive genetic profiling of CM1 becomes plausible. Larger studies may reveal converging pathways or alterations underlying CM1. Additionally, while most studies have focused on variants in protein-coding genes, noncoding variants may also play a role in gene regulation and likely contribute to CM1. Proposed developmental and evolutionary mechanisms in CM1 pathogenesis also suggest that changes in gene regulation are likely to be important. Though this has begun to be studied with RNA expression [19,103], there is much to be learned by expanding this to the transcriptome as a whole, as well as to other assays such as studies on epigenetic features and protein expression, which may yield additional mechanistic and functional insight.

Gene regulatory changes also tend to be tissue-specific. Recent studies have noted differences in alterations between dura and blood [19]. Therefore, we and others propose that exploring other tissues, such as bone and brain tissue in cases where resection is indicated, may yield further insights into CM1 genetics [165]. For example, studying bone is particularly important for insight into molecular mechanisms that may underlie posterior fossa abnormalities, and bone samples may be readily available for patients who undergo suboccipital craniectomy [165]. Of note, as new studies are developed, keeping detailed clinical records to accompany genetic profiles will also be important to account for different CM1 presentations. It will also be crucial to include patients of diverse ancestries and ethnicities to ensure that linked genes are represented across a wide range of genetic backgrounds.

## 6. Conclusions

In this review, we have discussed three broad classes of CM1 based on anatomy and linked them with genetic lesions, including posterior fossa-linked, macrocephaly-linked, and connective tissue disorder-linked CM1. Recent studies have provided significant headway into understanding the genetics of CM1, though larger studies and complementary technologies and methodologies are needed. Furthermore, as studies provide an increasing number of potential associations, approaches to understanding the functional consequences of gene variants and associations will also be needed [166]. We anticipate that we will continue to improve our understanding of the genetics and etiology of CM1 in the coming years, and are hopeful that this will translate into an improved selection of treatments and better clinical care for CM1 patients.
